# Enhancing Endometrial Health in Assisted Reproductive Technology (ART): Evaluating Autologous Endometrial Cells and Platelets-Rich Plasma (PRP) via Hysteroscopic Injections

**DOI:** 10.7759/cureus.64068

**Published:** 2024-07-08

**Authors:** Priti Karadbhajne, Akash More, Hellen Y Dzoagbe

**Affiliations:** 1 Clinical Embryology, Datta Meghe Institute of Higher Education and Research, Wardha, IND; 2 Obstetrics and Gynecology, Acharya Vinoba Bhave Rural Hospital, Wardha, IND

**Keywords:** infertility therapy, instillations, biomarkers, endometrial volumetric analysis, endometrial receptivity

## Abstract

This review article examines the effectiveness of incorporating autologous endometrial cells and platelet-rich plasma (PRP) through hysteroscopic injections within the field of assisted reproductive technology (ART). This study assesses the outcomes of these injections on the susceptibility of the endometrium, the dynamics of the uterus, and the frequencies of pregnancy in individuals with refractory thin endometrium. Based on a complete examination of several trials, it becomes apparent that autologous PRP injections provide encouraging turnouts in augmenting endometrial thickness, raising endometrial receptivity, and, in the end, raising chances of being pregnant and successful delivery. The research highlights the promise of autologous PRP and minimally changed endometrial cellular treatments in enhancing outcomes in ART, especially for people who have had problems with implantation. This article gives a whole evaluation of the medical use of and upgrades regarding the utilization of infusions of PRP and autologous endometrial cells under hysteroscopic control to deal with infertility issues related to endometrial health through the synthesis of contemporary studies.

## Introduction and background

The receptivity of the endometrium is an essential element in the effectiveness of assisted reproductive technology (ART) treatments, substantially impacting the implantation of embryos and the subsequent effects of being pregnant. The complex molecular and cell-mediated methods that alter the receptivity of the endometrium are of utmost significance for the optimization of fertility treatments and the development of successful cycles of ART [1]. The endometrium, a tissue that goes through cyclic hormonal modulation, undergoes a chain of molecular changes during the implantation period to offer good surroundings for the attachment of the embryo [2]. Continuous endometrial volumetric analysis has notably transformed the evaluation of endometrial receptivity in ART cycles, presenting a radical assessment of the endometrium's receptivity stage all through the phases of the treatment technique. There are biomarkers that have been recognized to be effective indicators of receptivity in endometrial patients, notably leukemia inhibitory factor (LIF), integrin, glycodelin, and homeobox A (HOXA); these biomarkers strengthen the assessment of receptivity that should be applied in ART [3]. With respect to the factors that cause implantation failure, broad-based strategies that could improve implantation include endometrial scratching, hormonal augmentation, and a transfer schedule that could increase the pregnancy rate and decrease implantation failure rates among affected patients.

Concerning the effectiveness of ART, it is very important to apply traditional ART protocols to address patient need(s) and/or requirement(s), which can even improve the probability of satisfactory embryo implantation and a satisfactory gestational rate. By explaining the complicated interplay of molecular, cell, and immunological tactics influencing endometrial receptivity, this evaluation seeks to offer a complete assessment of the relevance of platelet-rich plasma (PRP) and autologous endometrial cells (AECs) under hysteroscopic control in ART in terms of endometrial receptivity and its implications for clinical practice [4]. ART has appreciably transformed the domain of infertility therapy, providing optimism to several couples going through difficulties in conceiving. PRP and AECs injected underneath hysteroscopic management have emerged as a feasible route for a few of the modern procedures being explored to boost the effectiveness of ART. This innovative technique includes the right management of AECs and PRP without delay into the uterine hollow space to enhance endometrial receptivity and finally enhance the outcomes of ART remedies.

The implantation of PRP in ART under hysteroscopic control with AECs is principally based on the restorative properties of these materials. PRP, as mentioned earlier, has several advantages: it can promote the growth of new cells, which are essential for the growth of the endometrial layer, as well as facilitate the formation of new blood vessels and regeneration of new tissue, which are vital for thickening the endometrium and making it receptive [5,6]. Research findings suggest that the use of this particular approach can yield exquisite improvements in uterine hemodynamics and endometrial thickness and, in the long run, improve the rate of pregnancy amongst individuals recognized with refractory lean endometrium [7]. Cell development and angiogenesis can be stimulated with the aid of autologous PRP [8]. For example, it enhances the motion and attachment of endometrial stromal cells in a laboratory setting [9]. The process involves the discharge of growth elements, which can be stored in platelet granules and launched after platelet activation. Even though PRP infusions into the uterus hollow space are a minimally invasive method with benefits [10], these treatments, referred to as "installations," are unable to offer the endometrium's basal layer with sizeable concentrations of growth factors (GFs), in particular when their amounts are restrained to at least 1-2 mL.

It is impossible to magnify the importance of endometrial receptivity in ART, as it is a vital factor in deciding the outcome of ART treatments like in vitro fertilization (IVF) and embryo transfer. A woman's endometrium is at its peak ready state for embryo implantation within the time frame at some point of her menstrual cycle, referred to as endometrial receptivity. For a successful embryo to attach and finally establish a pregnancy, this time frame is essential [1]. Hence, good endometrial receptivity is needed for the effectiveness of ART treatments. Pregnancy success requires an interaction between the endometrium and the embryo for the duration of implantation [11]. In particular, the presence of some types of molecular characteristics in affected endometriosis tissues of women may also disrupt the embryo-endometrium dialogue, which threatens implantation [12]. However, studies have been unable to provide conclusive results related to the impact of endometriosis on implantation rates in ART cycles [13]. By determining fertility using techniques such as endometrial reactivity assay (ERA) and ultrasound assessment of endometrial characteristics, embryo transfer and early pregnancies can be synchronized to improve the chances of pregnancy [14,15].

According to previous studies, a low endometrial receptivity has been correlated with implantation failure or first-trimester abortions; therefore, the importance of this marker in achieving pregnancy through ART has been highlighted. These findings validate the relevance of endometrial receptivity - a factor that is critically dependent on the time as well as the notch of the month - an implication that reinforces the need for its exact measurement prior to the performance of ART. Various processes, along with ultrasound examination, endometrial biopsy for histological dating, and molecular marker analysis, are applied to evaluate the receptivity grade and choose the perfect timing for embryo transfer, improving the chances of successful implantation and achieving conception. Despite advancements in ART strategies and technologies, rates of implantation in stimulated cycles stay unsatisfactory. Enhancing our understanding of endometrial receptivity and coordinating embryo transfer with the prime receptivity window are critical methods to enhance the implantation rate. Identifying techniques to pick out excellent embryos and test endometrial health without interrupting the sensitive technique of implantation is essential for reinforcing ART effects [16].

The complicated interplay between the embryo and endometrium in the course of implantation includes a complicated communication that starts early in oocyte maturation. By reading the hormonal and molecular components concerned with this interaction, researchers hope to forecast endometrial receptivity or embryo superiority to upscale implantation rates in ART methods [17].

## Review

Autologous cell therapy (ACT) in ART

**Figure 1:**
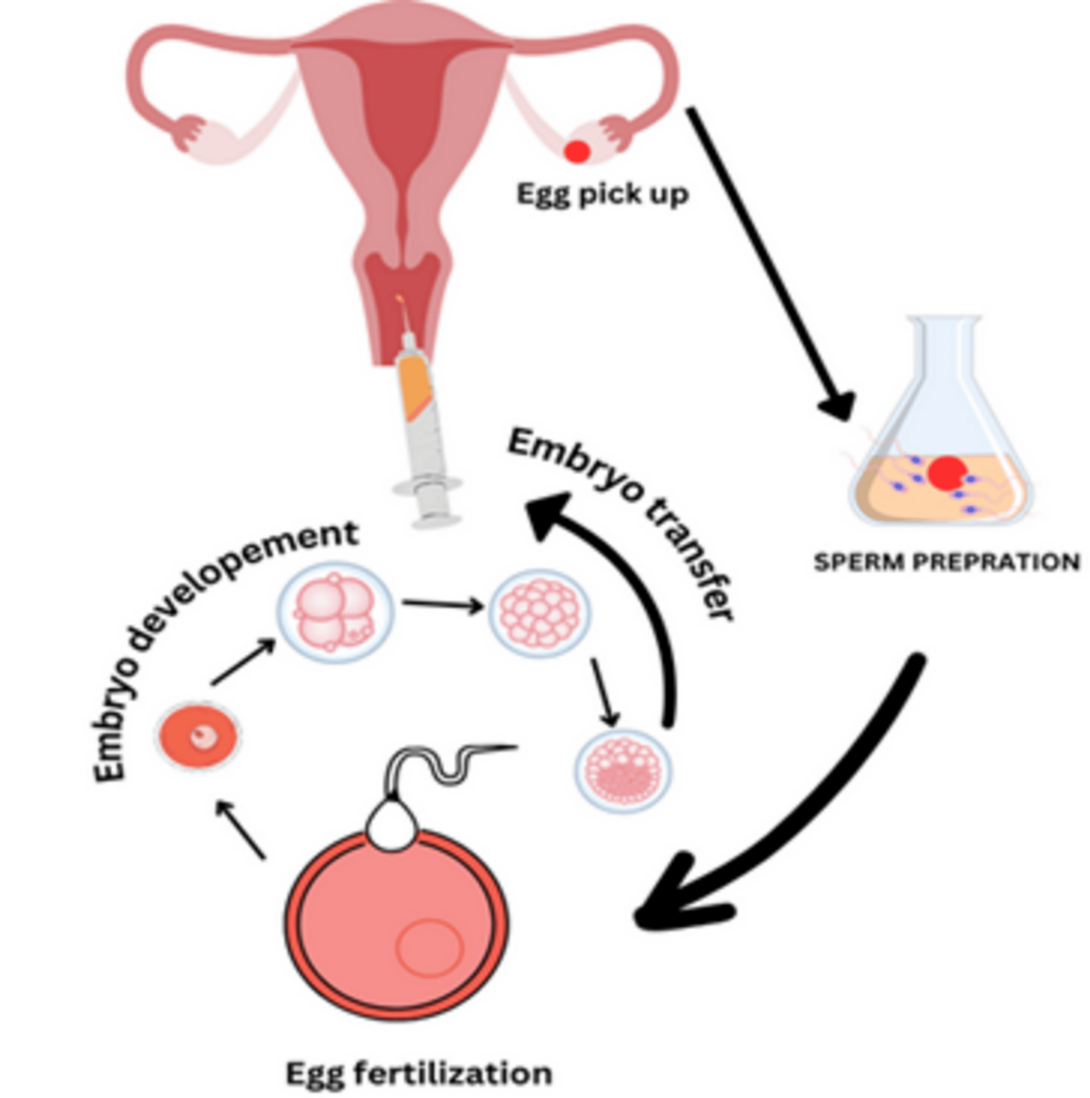
Assisted reproductive technologies for infertility The author created this figure using the Canva platform.

ACT has been researched in several fields, including ART therapy, blood transfusions, and treatment of cerebral palsy and acute myocardial infarction (AMI). In the sphere of ART, ACT is utilized as a type of adult development assistance, aiming to strengthen a man or woman's capacity to address everyday life and achieve self-alertness [18]. When receiving blood transfusions as part of ACT, the affected person is given both compatible donor blood and their blood as well [19]. The safety and viable development of motor features of autologous mononuclear cell remedy using bone marrow-derived cells or umbilical cord blood in the treatment of cerebral palsy has been studied [20]. In the setting of AMI, autologous bone marrow-derived mononuclear cells have been employed, but the use of autologous serum for cell preservation requires careful attention to decrease clotting and maintain cellular viability [21]. Figure 1 illustrates ART for infertility.

In the ART era, ACT refers to using an affected person's cells for medicinal purposes alongside reproductive remedies. This new method intends to take advantage of the regenerative potential of autologous cells to reinforce the success of ART treatments such as IVF and embryo transfer. By leveraging the regeneration traits of the patient's cells, ACT provides a tailored and probably more successful therapy alternative within the field of reproductive medication [22].

An article reported a case study that looked at a 35-year-old female who had infertility due to impaired or suboptimal tissues, including the endometrium. The affected woman underwent reconstructive procedures; these procedures involved autologous and intraovarian mesenchymal stem cell (MSC) and PRP administration. Regenerative clinical approaches, including the use of ACT, have the potential to boost IVF effectiveness and aid in the means of fertility treatments, which helped this woman achieve a successful pregnancy after the failed courses of IVF [23].

ART provides hope to couples who are experiencing infertility issues by extending a bias for the treatment options that are more appropriate for modification by certain requirements and circumstances. These have significantly transformed fertility procedures and preserved them through inventive methods aimed at optimizing efficiency and solving different causes of infertility.

Autologous stem cells

The kinds of stem cells are hematopoietic stem cells (HSCs) and MSCs, which are the frequent kinds of autologous cells utilized in medical operations [24]. HSCs are precursors of blood-forming cells and play a vital role in replenishing the blood cell population. Hematologic malignancies, including leukemia and lymphoma, are the ailments that these cells frequently treat via HSC transplantation (HSCT). On the other hand, MSCs are adult cells that can change into several types of cells, such as bone, cartilage, and fats. These cells function in immunomodulatory and anti-inflammatory capabilities, making them precious for regenerative medicinal applications [24].

Autologous cell remedies contain the harvest and reintroduction of an affected person's cells back into their body. Adipose tissue, bone marrow aspirate and concentrate, α-2 macroglobulin, platelet lysate, PRP, interleukin-1 receptor antagonists, and cultured cellular injectates are some examples of autologous products [19]. For patients with diabetes suffering from no critical limb-threatening ischemia (NO-CLTI), ACT may be derived from bone marrow, utilizing diverse isolation strategies or inspired peripheral blood [25]. ACT using umbilical cord blood or bone marrow-derived mononuclear cells has been tested for the treatment of cerebral palsy [21]. With continuing scientific trials and successful transplants, human induced pluripotent stem cells (iPSCs) have shown promise for autologous stem cell-based therapies [26]. Tumor stem cells and different autologous cells taken from patients' very own tissue have been studied for use in urological tissue engineering and regeneration [27].

Types of Autologous Cells

HSCs are crucial for the recuperation of hereditary hematologic, metabolic, and immunologic conditions. After transplantation, they can reconstruct and preserve a functioning hematopoietic system for a prolonged time frame [28]. Individuals with compromised or non-functioning immune structures or bone marrow can go through HSCT to reinstate blood cell formation. Two methods are used: autologous and allogeneic [29]. HSCT is mechanically utilized in the remedy of infantile leukemia, and obtaining minimal residual ailment-negative state prior to transplantation and proper management of post-transplant problems are important variables for improved effects [30]. Glioma stem cells (GSCs) have been tested for therapeutic use, identification of stem cells, and effective employment in glioma remedies [31]. The production of all sorts of blood cells is attributed to HSCs, which have pluripotency and self-renewal abilities. They have been used for decades as the remedy for many malignancies and blood complications [32].

HSCs are important for rebuilding the blood-cell populace through a system known as hematopoiesis. They give rise to several blood cell types, consisting of lymphoid (e.g., T cells, B cells) and myeloid (e.g., monocytes, neutrophils). Since one can obtain HSCs from peripheral blood, bone marrow, and umbilical cord blood, they are good sources for treatments, including stem cell transplants and regenerative medicine [33]. HSCs are very important and are required in the human body to guarantee a population of blood cells and immunological features. Due to their unique characteristics and functions, as well as their origin, they are considered valuable organs in regenerative medicine and stem cell therapy [33]. It is imperative to comprehend the biology and clinical application of HSCs in an effort to develop therapies for several hematological diseases further and to understand their impact on patients. Subsequently, by exploring this highly complex domain of HSCs, researchers, and therapists can open up new possibilities for managing blood-related conditions, degenerative disorders, and immune deficiency diseases and create a path for future restorative procedures and streamlined strategies for individualized care.

Peripheral Blood Stem Cell Transplant (PBSCT)

The use of PBSCT is a feasible therapeutic approach for individuals diagnosed with hematological malevolence. Human leukocyte antigen (HLA) matched, related, or unrelated donors, as well as haploidentical related donors in the absence of an HLA-matched donor, utilize this therapy approach. Post-transplant cyclophosphamide (PTCy) has been shown to reduce the occurrence of graft-versus-host disease (GvHD) and non-relapse mortality (NRM) in the haploidentical peripheral blood stem transplant (haploPBSCT) [34]. Furthermore, the incorporation of corticosteroids (CSs) into GvHD prevention protocols in PBSCT no longer seems to contribute to improving effects [35]. The composition of the graft, which includes the number of B cells, NK cells, and T cell subsets, can affect the result of PBSCT when received from unrelated people [36].

PBSCT, also known as "peripheral stem cell support," encompasses the retrieval of blood-forming stem cells from the peripheral blood. The replenishment of the blood cell populace is an essential function of stem cells, which might be regularly diminished through most treatment plans for cancer, like chemotherapy or radiation, in addition to several blood-related situations like leukemia, lymphoma, and multiple myeloma [37]. PBSCT is frequently done since it is convenient and less intrusive than the traditional bone marrow harvest. The treatment has validated advanced effects in terms of HSC formation and is used for the control of various medical ailments, including leukemia and multiple myeloma. Nevertheless, allogeneic PBSCT is, by and large, linked to more complications as compared to autologous PBSCT, because of disparities between the donor and recipient [38].

PBSCT serves a widespread role in restoring wholesome blood cell formation in individuals undergoing cancer treatments or handling blood-related issues. Understanding the method, varieties, utilization, and implications of PBSCT is vital for healthcare specialists collaborating in HSCT. Further examination and breakthroughs in this area can reason enriched outcomes for those requiring stem cell transplantation. By delving into the complexity of PBSCT, researchers and clinicians can better their expertise and practices in using this method effectively for treating a spectrum of hematologic illnesses.

MSCs

**Figure 2:**
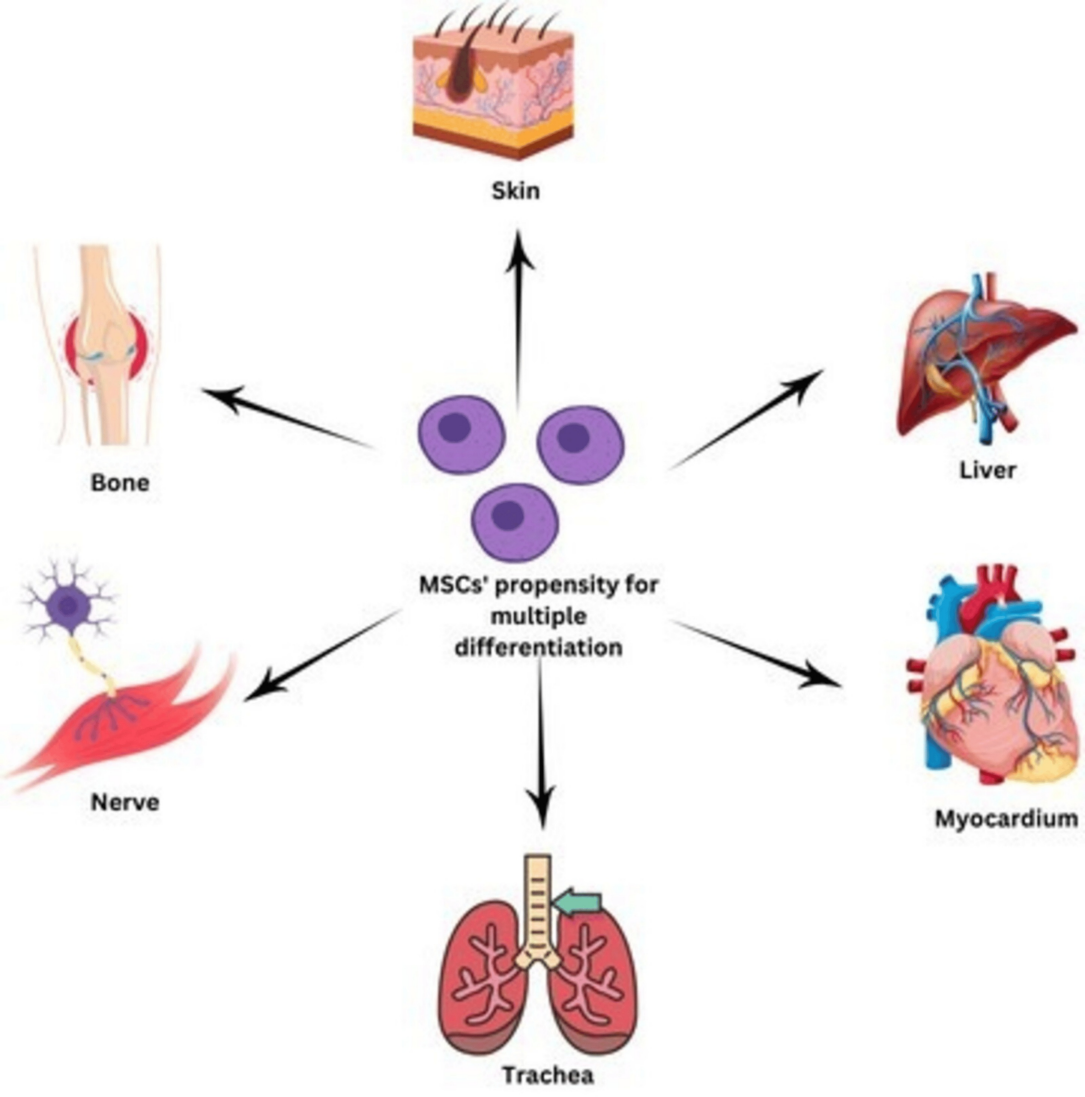
MSCs propensity for multiple differentiation MSCs: mesenchymal stem cells The author created this figure using the Canva platform.

Multipotent stromal cells with a high proliferative capacity, such as MSCs, can turn into numerous types of cells. They exhibit immunosuppression and can flow to injured tissues. MSCs have been established to have therapeutic capacity in experimental models and scientific trials, making them an alternate method to traditional treatment techniques [39]. MSCs have been applied to treat neurological conditions like Parkinson's disease, multiple sclerosis, and Alzheimer's disease. They can be extracted from different body tissues [40]. However, there are discrepancies throughout MSC resources, which could restrict their powerful utilization [41]. Because of their trophic qualities, MSCs have the potential to regenerate surrounding tissues and repair several cell populations [42]. Furthermore, because of their demonstrated protection and effectiveness in medical trials, adult telomerase-positive stem cells (PSCs) have been advocated as a supplement to regenerative remedies [43]. They are beneficial gadgets in clinical research and regenerative medicine because of their regenerative traits [44]. Figure 2 represents MSCs' propensity for multiple differentiation.

Cord Blood Transplant

HSCs are isolated from the umbilical cord after the delivery and stored in cord blood for later use in clinical processes [45-47]. Through cord blood transplantation (CBT), these stem cells have proven promising effects in treating immunological deficits, hematological malignancies, and genetic anomalies [48,49]. Wider HLA disparity tolerance, a decreased possibility of GvHD, and intensified value in lifestyles-associated survival are just a few compensations for the usage of cord blood as a source of donor cells for transplantation. Additionally, cord blood units may be stored for long periods without losing their viability, making them an able resource for future remedies. Cord blood banks have been modified to maintain stem cells for clinical and research purposes.

To aid in the regeneration of wholesome blood cells in a patient, umbilical cord blood stem cells are infused into the affected person's circulation all through cord blood transplant. When a related or unrelated donor who suits the recipient is not accessible, this kind of transplant is especially helpful. Cord blood, rich in stem cells, can be extracted from the umbilical cord after a child's delivery and kept for later use. The preserved cord blood may be utilized for transplants to treat ailments like aplastic anemia, hereditary blood abnormalities, and some cancers. After the cord blood cells are thawed and infused into the patient's bloodstream, a process referred to as engraftment occurs in which the cells flow to the bone marrow and start to produce healthy blood cells. Although there are gains to cord blood transplants, including a decreased threat of GvHD, the engraftment procedure can be time-consuming since there are fewer stem cells in cord blood donors than in mature donors. Individuals receiving cord blood transplants may additionally come across transient adverse outcomes such as infections, mucositis, and exhaustion; however, the lasting effects may also fluctuate [50].

Mechanism of Action for Autologous Cells

**Figure 3:**
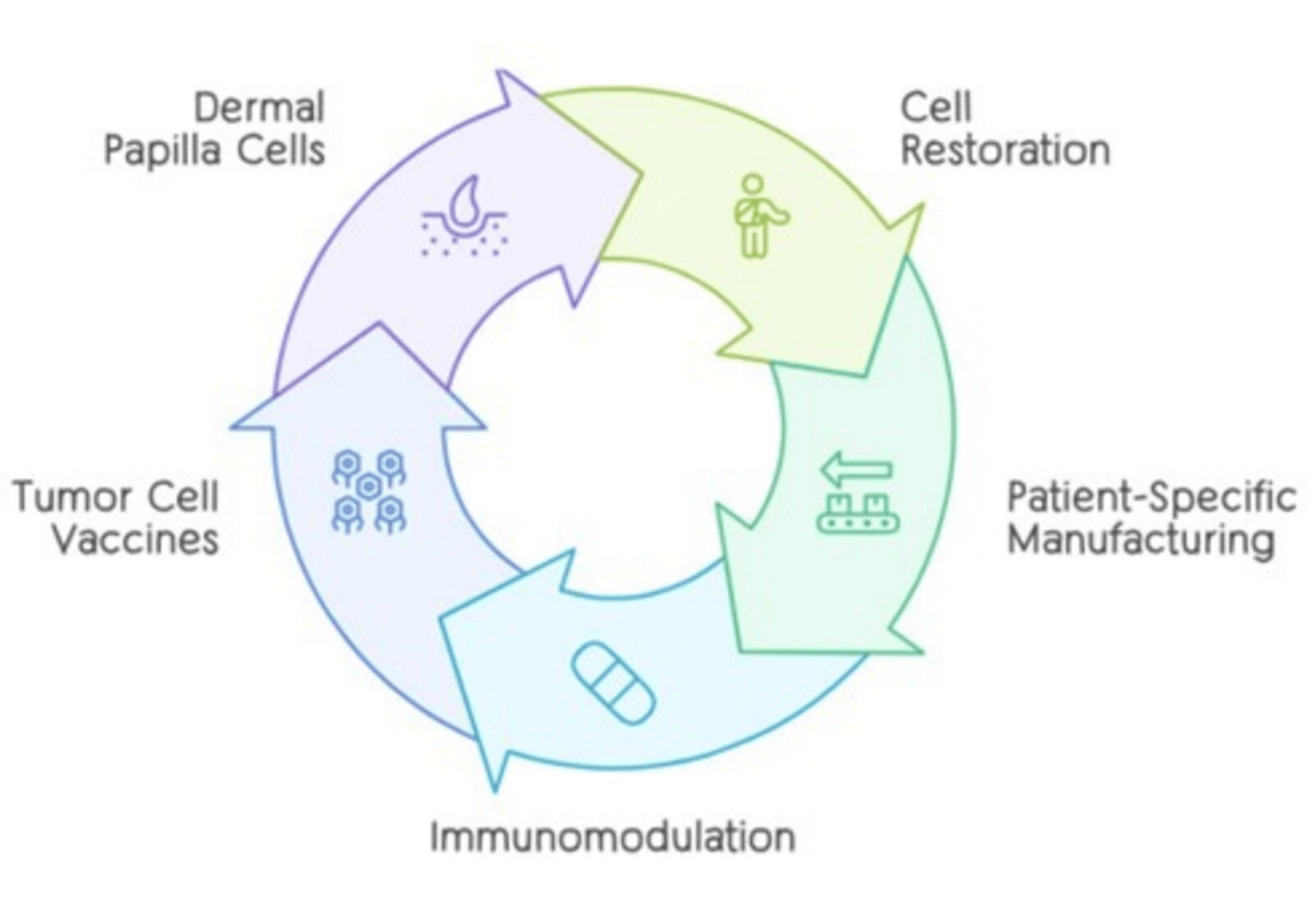
Key processes mechanism of action for autologous cells The author created this figure using the Canva platform.

The method of harvesting and reintroducing a patient's cells or tissues for medicinal functions is called ACT. These treatment plans have been researched in several fields, such as plastic surgery, orthopedics, and dermatology. Dermatology has made use of autologous substances such as adipose tissue, platelet lysate, and PRP [51]. Autologous tolerogenic dendritic cell therapy (DC therapy) has been created to deal with hemophilia by inducing immunological tolerance to either Factor VIII (FVIII) or Factor IX (FIX) [19]. In patients affected by intense left ventricular (LV) failure, autologous cardiac progenitor cells (CPCs) have demonstrated promise in enhancing coronary heart function [52]. Autologous cell remedies function by utilizing the affected person's cells to enhance tissue transformation and angiogenesis, control the discharge of GFs and cytokines, and boost restoration [53]. These approaches offer the value of being biocompatible and without risks of hypersensitive reactions and transfer-related infections. GFs that help tissue regeneration and repair, for example, are discovered in PRP, and growth elements that might be soluble and secreted from autologous dermal papillae cells act on surrounding epithelial matrix cells to stimulate the growth of hair. When autologous stem cells are transplanted for bone marrow repair, for example, they can change into a couple of cells and substitute the body's damaged or non-functioning cells. Generally, autologous cell treatments use the affected person's very own cells to support immune regulation, tissue regeneration, and repair. This customized method of treatment may be more of a success than allogeneic cell treatment plans. Figure 3 explains the mechanism of action for autologous cells.

PRP injections in ART

ART has made PRP injections a doubtlessly effective way to boost endometrial receptivity and enhance pregnancy results. Derived from the patient's blood, PRP is extraordinarily concentrated in GFs and cytokines that are vital for tissue regeneration and repair. PRP injections are utilized in the context of ART to deal with situations such as implantation failure, low ovarian response, and thin/lean endometrium. PRP remedy has been confirmed in research to improve the potential for fruitful pregnancy IVF cycles, stimulate endometrial improvement, and increase endometrial receptivity [54]. PRP acts on the endometrial tissue via the interplay of growth elements and cytokines, promoting angiogenesis, regeneration, and cell proliferation. These factors, which encompass platelet-derived growth factor (PDGF) and vascular endothelial growth component (VEGF), assist in creating an environment that is conducive to the implantation and progress of embryos. Increased endometrial thickness - which is critical for successful implantation and pregnancy - has been related to PRP injections [55].

**Figure 4:**
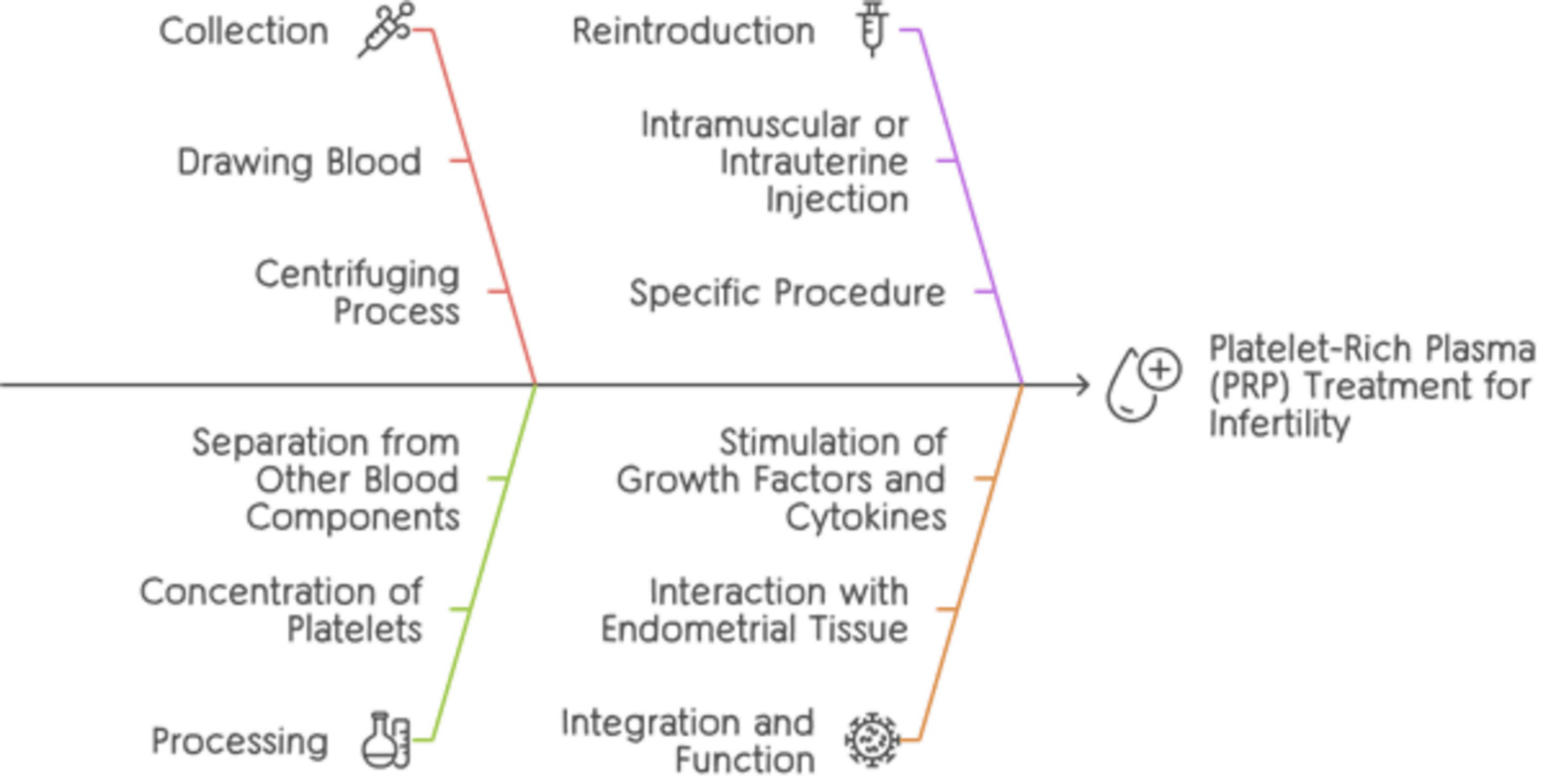
The mechanisms of action of PRP in ART ART: assisted reproductive technology; PRP: platelet-rich plasma The author created this figure using the Canva platform.

PRP has been proven in reproductive research to have a useful impact on follicular and endometrial development, which helps gestation. PRP has been shown in scientific trials to be effective in resolving problems, including inadequate endometrium growth in previous cycles and improved endometrial receptivity. Patients have demonstrated better outcomes in pregnancy after receiving PRP intrauterine injections, underscoring PRP's significance in enhancing the efficacy of infertility remedies [54]. Figure 4 gives an explanation of the mechanism of action of PRP.

Platelet counts of individuals vary, which highlights the significance of personalized therapy plans when using PRP in ART. There are many preparation strategies, consisting of leucocyte- and platelet-rich plasma (L-PRP) and pure PRP (P-PRP), which are each designed for specific clinical use. Variations in PRP preparation are supposed to maximize the product's bioactivity and improve its therapeutic advantages on embryo implantation and endometrial receptivity [54]

PRP Composition and Preparation

Important elements that affect PRP’s healing efficacy in a range of medicinal utilization consist of its processing and composition. PRP is a custom-designed therapy alternative since it is a preparation of platelet and growth components made from the affected person's blood. Peripheral blood should first be drawn from the patient. This is the first of several crucial tactics within the creation of PRP. After that, the blood is centrifuged to extract the liquid and cell components, concentrating the platelets and reducing the erythrocyte content material. Usually, there are spins involved inside the centrifugation process: the first spin separates platelets from RBCs and WBCs, and the second spin concentrates platelets even more, right into a layer containing white blood cells [56].

The formulation techniques and composition of PRP vary during research. PRP's composition varies depending on the practice approach used, and this consists of versions in platelet, leukocyte matter, and subtypes [57,58]. Higher baseline platelet concentrations bring about better platelet concentrations in PRP. Baseline platelet content in entire blood samples impacts platelet amount in PRP. Prepausally, PRP has platelet content that is usually higher than that of complete blood [58]. PRP's leukocyte, lymphocyte, and neutrophil ratios vary from the whole type of blood [59]. Compared to single centrifugation techniques, double centrifugation protocols produce greater platelets and yield less infection from RBCs and WBCs. The absence of standardization in PRP manufacturing and composition reporting makes it hard to evaluate trials and investigate efficacy.

Endometrial Receptivity and PRP's Effect

PRP has a remarkable impact on endometrial receptivity when ART is placed. PRP remedy has proven effective in endometrial thickness, embryo implantation, and success of clinical pregnancy in patients with thin endometrium, recurrent implantation failure (RIF), chronic endometritis (CE), and Asherman’s syndrome (AS) [60]. The mechanisms via which PDGF, VEGF, epidermal growth factor (EGF), transforming growth factor-beta (TGF-β), insulin-like growth factor 1 (IGF1), and other GFs and cytokines are present in PRP are answerable for their capability to augment endometrial receptivity. These factors include all those that support cell growth, division, and development, which improves endometrial receptivity.

PRP has been observed to beautify the proliferation of several endometrial cells, including epithelial cells, stromal fibroblasts, and mesenchymal stem/progenitor cells. Studies have shown more advantageous cell proliferation in human endometrial stromal cellular lines and key stromal cells following PRP treatment. Additionally, PRP has been related to the extra expression of adhesion molecules, improved stem cell appeal, better endometrial cellular migration, and heightened tiers of HOXA10, a crucial hallmark of endometrial receptivity. Moreover, PRP influences endometrial receptivity due to its anti-inflammatory and anti-microbial traits. The nuclear factor kappa-B (NF-κB) is a major regulator of irritation, and PRP inhibits it from generating surroundings that are conducive to tissue and cell growth and proliferation. Improvement in amounts of successful pregnancies, including extended endometrial thickness, number of live births, and gestational outcomes in individuals with thin endometrium and different reproductive troubles, were related to intrauterine PRP treatment [54].

GFs and Cytokines

GFs and cytokines are protein regulators that manipulate the formation of cells and have widespread features in numerous tissues and physiological processes. They are created within the tissues wherein they act and are concerned with controlling the self-renewal of stem cells, cellular proliferation, differentiation, motility, and survival. Inappropriate production or reaction to GFs can result in sicknesses of cancer, autoimmune sicknesses, and inflammatory illnesses. In most cancers, growth aspect receptors may be altered to supply hyper-responsive versions, inflicting tumor improvement and spread. Treatment plans for cancer contain GF antagonists or inhibitors to restrict tumor cellular proliferation and infiltration. Additionally, GFs and cytokines have potential therapeutic and diagnostic applications, and the introduction of world standards for their power and characterization is ongoing [61].

Vital insights into the GFs and cytokines present in PRP and their usefulness in many scientific packages. PRP is rich in bioactive chemical compounds, consisting of GFs and cytokines, that play crucial roles in tissue recuperation, regeneration, and management of inflammatory responses. PRP consists of some crucial boom factors, which include hepatocyte growth factor (HGF), TGF-β, IGF1, fibroblast growth factor 2 (FGF-2), vascular endothelial growth factor (VEGF), PDGF, and EGF [62,63].

Angiogenesis and Endometrial Regeneration

Endometrial regeneration and angiogenesis are intently connected approaches. Numerous investigations have looked into the possibility of using diverse strategies to inspire endometrial regeneration and induce angiogenesis. One study evaluated the utility of an all-in-one smart dressing (ASD) that incorporated angiogenic purposeful substances and several biological factors to stimulate angiogenesis and neuronal regeneration simultaneously [64]. An angiogenic hydrogel microsphere VEGF was made in exceptional research and used to treat a skinny endometrium [65]. There are principal forms of endometrial angiogenesis: non-sprouting angiogenesis, often called intussusception, and sprouting angiogenesis. Sprouting angiogenesis encompasses procedures including endothelial cell proliferation, migration, invasion into the stroma, and tube creation. Conversely, non-sprouting angiogenesis is characterized by the proliferation of endothelial cells inside capillaries, which can result in capillary fusion and splitting or transcapillary pillars splitting arteries. These activities are strictly regulated by using the stability of inducers and inhibitors of angiogenesis.

VEGF is a crucial angiogenic component involved in endometrial vascular enlargement and transformation. Research has proven that the angiogenic capacity of the human endometrium varies cyclically over the direction of the menstrual cycle. Enhanced expression of VEGF at some point in stages of the cycle is associated with unique vascular alterations, which can be required for tissue reconstruction, vessel elongation, spiral arteriole coiling, and vasculature preservation. The dynamic modulation of VEGF and different angiogenic factors contributes to the cyclical nature of endometrial regeneration and vascular growth [66]. Angiogenesis is an important step in endometrial regeneration that guarantees suitable tissue development, vascularization, and receptivity for embryo implantation. The complicated interaction among angiogenic factors like VEGF and the complex mechanisms of vascular expansion in the endometrium underline the relevance of understanding normal endometrial angiogenesis for resolving reproductive diseases and maximizing fertility effects.

Autologous cell and PRP combination therapy

Combination remedies involving autologous cells and PRP have promising outcomes in lots of applications. Studies have mentioned the safety and efficacy of combining autologous derived drug treatments inclusive of PRP, lipofilling, or stromal vascular fraction (SVF) with scientific devices for scar remedy, resulting in better scar eminence and feature [67]. Precision blood component separation and the advent of PRP with a defined composition are made possible by utilizing the improvement of a reliable clinical system bearing the CE mark, which is meant for the standardized practice of PRP and different autologous biologics [68]. Combining MSCs with autologous PRP has upheld potential within the management of gastric leaks after sleeve gastrectomy by accelerating the recovery [69]. Atrophic pimple scars have been efficiently handled with the use of autologous fats, stem cell-derived microspheres, and PRP transplantation, which has considerably enhanced the inclination of patients to results and skin clarity [70]. It has also been validated that the usage of PRP along with adipose-derived stem cells (ADSCs) can stop human dermal fibroblasts from aging by boosting the expression of the protein retinoblastoma (Rb) [71].

Numerous illnesses may be dealt with through the use of combined therapy regarding autologous cells and PRP. This novel approach has demonstrated the capacity to treat lots of illnesses, consisting of solid vitiligo, elbow tendinopathy, knee osteoarthritis (KOA), skin problems like burns, scars, and ulcers, and orthopedic troubles like tendinopathy, ligament damage, muscle traces, and cartilage injuries. Additionally, this remedy is being researched for its capacity in most cancers remedy, cardiovascular illnesses, and neurodegenerative sicknesses, such as Alzheimer's, and for general health and well-being promotion. Combining autologous stem cells with PRP reduces the risk of immunological rejection, improves therapeutic effects, and expedites healing with the aid of repairing some tissues or organs [72,73].

In numerous dermatological packages, the blended therapy of autologous cells and PRP has attained an encouraging feat for treating skin conditions. Research has verified its effectiveness in treating several disorders, including androgenetic alopecia, refractory cutaneous ulcers, pimple scar treatment, and pores and skin rejuvenation. Notably, the most powerful evidence has been recognized in androgenetic alopecia and face skin rejuvenation. While the remedy has been validated to be beneficial in numerous dermatological disorders, the relative dearth of extremely superior and large randomized controlled trials (RCTs) restricts ordinary use. Combining autologous cells and PRP has shown promise in treating skin issues, albeit the data are not robust. Some studies have determined substantial improvements in patient satisfaction, skin smoothness, scar repair, and hair recuperation [74,75].

Rationale for Combined Approach

The combination of MSCs and PRP aims to enhance chondrocyte regeneration to restore the functionality of cartilage tissue, reduce inflammation in synovial fluid, and reduce the symptoms of KOA since it identifies the central cause of KOA. Although both MSCs and PRP independently showed positive outcomes in treating KOA and combinations of both have never previously been tested, research is still needed to determine the safety and efficacy of this technique due to the interaction between the two treatments [76].

The biological effects of combining PRP and MSCs are the result of a mechanism of action involving tissue repair and regeneration. While MSCs possess the potential for cell repair and renewal in some way, they are also capable of developing into a number of cell types, such as chondrocytes or osteoblasts, which are crucial for fixing damaged tissues such as cartilage. PRP provides the opportunity to apply a pure concentrate of GFs (VEGF, PDGF) that may be essential for tissue repair and regeneration [77].

When MSCs and PRP are mixed, they promote the proliferation, differentiation, and migration of cells in a healing manner. This combination treatment seeks to accelerate wound recovery, enhance tissue regeneration, reduce infection, and alleviate symptoms associated with illnesses such as osteoarthritis, bone abnormalities, muscle accidents, and skin issues [78]. Stem cells derived from the mesenchyme or bone marrow, when combined with PRP, promote cell proliferation, differentiation, or migration, particularly during the healing process. This combination treatment aims to quickly heal wounds, improve tissue remodeling, prevent infection, and thereby relieve diseases such as osteoarthritis, bone irregularities, muscle necrosis, and skin disorders. The biological potential of the interplay between MSCs and PRP is the ability to create favorable conditions for tissue restoration by stimulating cell proliferation, enhancing angiogenesis, and regulating inflammation, which leads to better outcomes in many restorative applications. [79].

Synergistic Effects of Autologous Cells and PRP

The synergistic advantages of autologous cells and PRP entail a combined therapy method that harnesses the regenerative and restoration houses of everything. Autologous cellular treatment, utilizing an affected man or woman's cells and PRP, wealthy in GFs, work collectively to promote tissue restoration, regeneration, and wound recovery. Studies have demonstrated that PRP can increase the proliferation and differentiation of numerous cells, while autologous cells like MSCs can distinguish unique cellular types important for tissue restoration [80]. When combined, this treatment produces a fantastic environment for tissue regeneration through stimulating cellular proliferation, growing angiogenesis, regulating infection, and improving wound restoration outcomes. The synergistic results of autologous cells and PRP have been recognized in several fields for face rejuvenation, pores and skin problems like burns and scars, orthopedic accidents, and even some cancer remedies. This blended method gives tailored remedy options with the capacity for stronger results in tissue recovery and overall health and well-being [81].

In 2019, Sfakianoudis et al. treated a patient with RIF through the intrauterine injection of autologous PRP and specific MSCs derived from menstrual blood. It was then concluded that the pregnancy and the live birth rates were significantly greater in the treatment group than in the control group [82]. Aghajanova et al., in 2018, reported their findings, which showed that the treatment of autologous PRP in the culture medium during IVF was effective in improving the maturation of the embryos and thereby increasing the implantation rate. Based on these findings, it can be suggested that a rational approach involving the use of autologous PRP and MSCs could promote the enhancement of endometrial receptivity, which is important for patients receiving ART [81,82]. However, large-scale clinical trials that include a greater number of subjects and comply with universally accepted research protocols are still needed to confirm the effectiveness and lack of harm of this intervention.

Safety and ethical considerations

Concerning targeted treatments and personal approaches, ACT is supposed to use the user’s cells, and PRP remedies obtained from the patient’s blood are not far from immune rejection points. However, ethical considerations highlight the understanding of the type of treatment that a patient is willing to undergo, any form of danger or risk involved, and the potential outcomes [82]. In fact, before and during the preparation and application of autologous cells together with a PRP, certain conditions must be met such that the therapy is as safe as possible. Issues relating to contagious diseases, such as HIV, are also important concerns whenever these cells are treated in terms of safety and the process to be followed. In addition, the possible dangers of activating further than the least impact on cells, such as transduction or ex vivo multiplication, to practice the necessary changes must be critiqued to counteract the worst [82].

## Conclusions

Considering the various published papers and literature on the effects of hysteroscopic injection of PRP and endometrial cells for ART treatment, it is evident that it has had some positive impact on fertility care. The series of these “advanced” techniques may have some significant benefits, such as better endometrial receptivity, improved probabilities of embryo implantation, and superior ART performance. It is perhaps beyond the scope of this level of research to definitively affirm or deny these results; however, the results are promising and would likely need further research, such as odd studies and long-term efficacy and safety clinical trials, to ensure the prospects of excellent reproductive effects. Hence, the advancement of reproductive medicine is still growing, and PRP injection and AEC transposition through hysteroscopy seems to be one of the most important steps in improving fertility and thus enhancing ART therapy for patients.
